# Supplementary material for: Topologically associating domains are ancient features that coincide with Metazoan clusters of extreme noncoding conservation
Source: Nat Commun. 2017 Sep 5;8:441. doi: 10.1038/s41467-017-00524-5 (PMC5585340; doi:10.1038/s41467-017-00524-5)
Supplement: Supplementary file 1 — Supplementary Information [file 41467_2017_524_MOESM1_ESM.pdf]

Description of Supplementary Files

File Name: Supplementary Information
Description: Supplementary Figures, Supplementary Tables, Supplementary Methods and Supplementary References

File Name: Supplementary Data 1
Description: GRB predictions generated using comparisons of hg19-galGal4 (70%/50bp).

File Name: Supplementary Data 2
Description: GRB predictions generating using comparisons and dm3-droMoj3 (96%/50bp).

File Name: Peer Review File

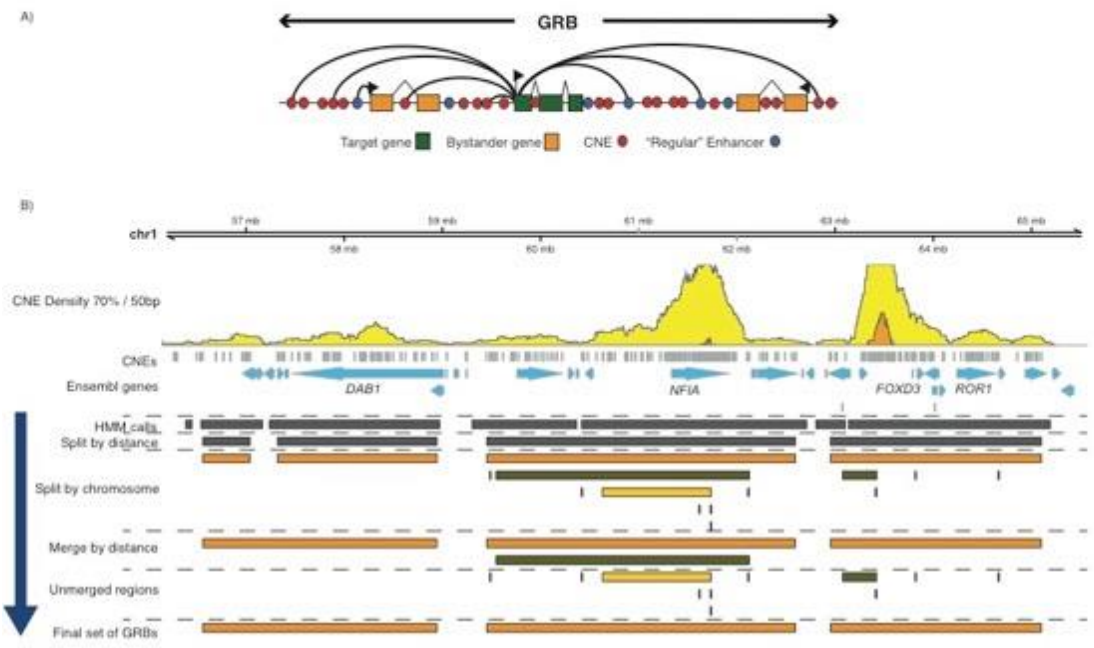

Supplementary Figure 1: Description of GRB model and schematic of the method for segmenting the genome into GRBs. A) Developmental genes are often found surrounded by arrays of conserved noncoding elements (CNEs), many of which act as long-range regulatory elements of this gene. In addition to this trans-dev gene under long-range regulation, a GRB can contain other genes known as bystander genes, which are not under this type of long-range regulation but contain CNEs within their introns which regulate the target gene of the GRB. The requirement to keep developmental regulators in *cis* with their regulatory elements results in the maintenance of synteny between CNEs, target genes and bystander genes over large evolutionary distances[1,2]. Bystander genes and target genes can also be under the regulation of other elements within the region that are not under such high levels of conservation. B) The region chr1:56136940-65569360 contains several distinct regions of CNE density overlapping with known developmental regulators (*DAB1*, *NFIA*, *FOXD3*). Our CNE clustering method (see Methods) splits the CNE density obtained from ANCORA into distinct regions by first segmenting the density using a HMM into regions of high and low density, and then clustering the CNEs together using the distance between them. This is followed by the application of various heuristics to generate a set of putative GRBs. These regions are split by the chromosome that they originate from in the query species, and merged with nearby regions if they are from the same chromosome and within a specified distance. While we expect a number of GRBs to be incorrectly segmented, the resulting set of GRBs appear to match, via visual inspection, the known GRB architecture at a number of loci.

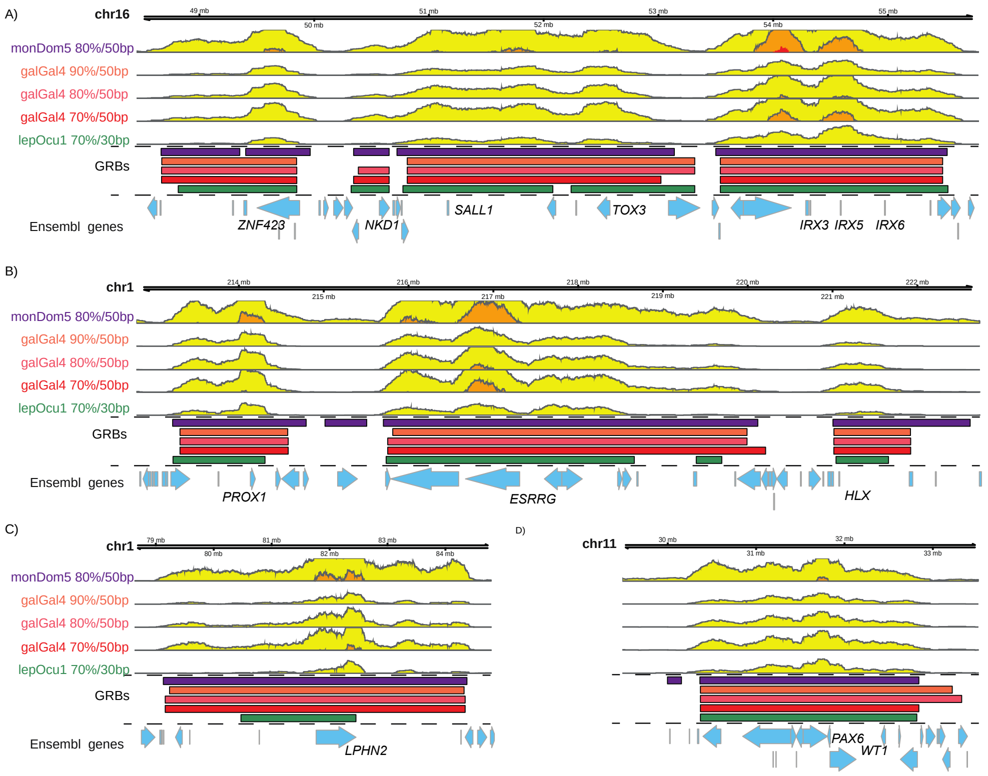

Supplementary Figure 2: The boundaries of GRBs are highly consistent regardless of thresholds or species. A number of loci in human are enriched for CNEs identifiable between evolutionarily distant species at various thresholds. For each set of CNEs, the corresponding CNE density is displayed as a horizon plot along with the corresponding set of putative GRBs for each comparison. A) A large chromosomal region in human (chr16: 48476700-55776880) contains several GRBs which are highly conserved over multiple evolutionary comparisons. The edges of the predicted *IRX3/5/6* GRB are highly concordant between all species and thresholds investigated, with the other GRBs in this region showing strong agreement in the majority of cases. The putative GRB identified, using comparisons with opossum and chicken, containing TOX3 and SALL1 is known to correspond to two distinct GRBs[3,4], however we are not able to separate them based using our clustering approach. B) A region (chr1:212820540-222701560) containing three known developmental regulators (*PROX1*, *ESRRG* and *HLX*) is segmented into three GRBs. In the majority of cases at this region at least one boundary of a GRB appears to be identified consistently over all comparisons. C) The GRB (chr1: 78652340-84766720) around *LPHN2*, a latrophilin involved in cell adhesion, has similar boundaries in comparisons generated using monDom5 and galGal4 whilst the GRB identified using lepOcu1 is much smaller. D) While it is known that the region (chr11:29500000-33500000) containing *PAX6* and *WT1* forms two separate GRBs[5], we are unable to distinguish between these regions based on CNE density alone. One edge of this GRB appears to be robustly identifiable in all comparisons with the other edge exhibiting some variation.

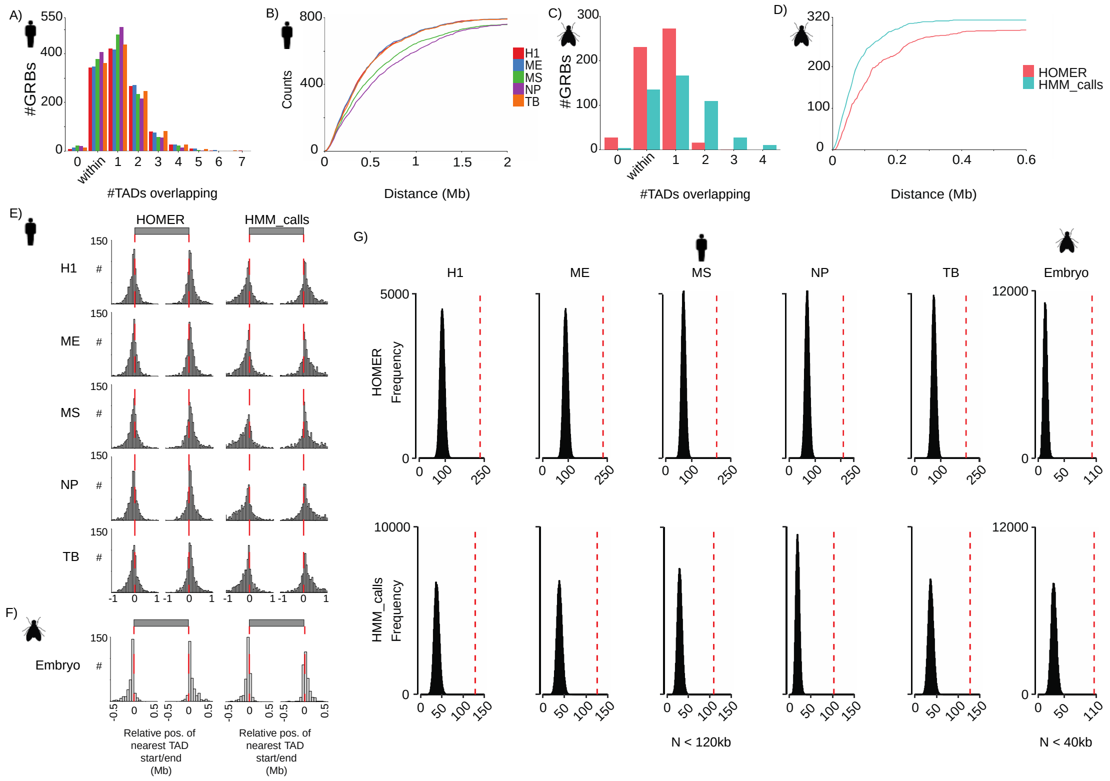

Supplementary Figure 3: The boundaries of GRBs predict the boundaries of TADs identified in multiple cell lineages A) A large number of hg19-galGal4 GRBs was found to be located within individual TADs (identified using HMM_calls) or overlapping only a single TAD, regardless of cell lineage. B) Cumulative distribution of distance to nearest TAD (HMM_calls) boundaries from GRB boundaries in different cell lineages considering both edges i.e. both the start and end position of a GRB lie within Xkb of the nearest TAD start and end. C). A large number of dm3-droMoj3 GRBs was found to be located within individual TADs (identified using HOMER and HMM_calls) or overlapping only a single TAD. D) Cumulative distribution of distance to nearest TAD (HOMER and HMM_calls) boundaries from GRB boundaries in *Drosophila* whole embryos considering both edges, i.e. both the start and end position of a GRB lie within Xkb of the nearest TAD start and end. E) Relative position of the nearest TAD start/end compared to the boundaries of hg19-galGal4 GRBs, using TADs identified in multiple cell lineages (H1-ESC (H1), mesenchymal stem cells (MS), mesendoderm (ME), neural progenitor cells (NP) and trophoblast-like (TB)). F) Relative position of the nearest TAD start/end compared to the boundaries of dm3-droMoj3 GRB, using TADs identified in Drosophila Hi-C data. G) Null distributions of number of GRBs with both edges lying within 120kb (for human centric) or 40kb (for *Drosophila* centric) across all cell lines and methods investigated (see Methods). Number of observed GRBs show as a red dotted line (see Supplementary Table 3). Given the null distribution the correspondence of our set of putative GRBs with TAD boundaries at these distances was significant across all comparisons (p<1e-5).

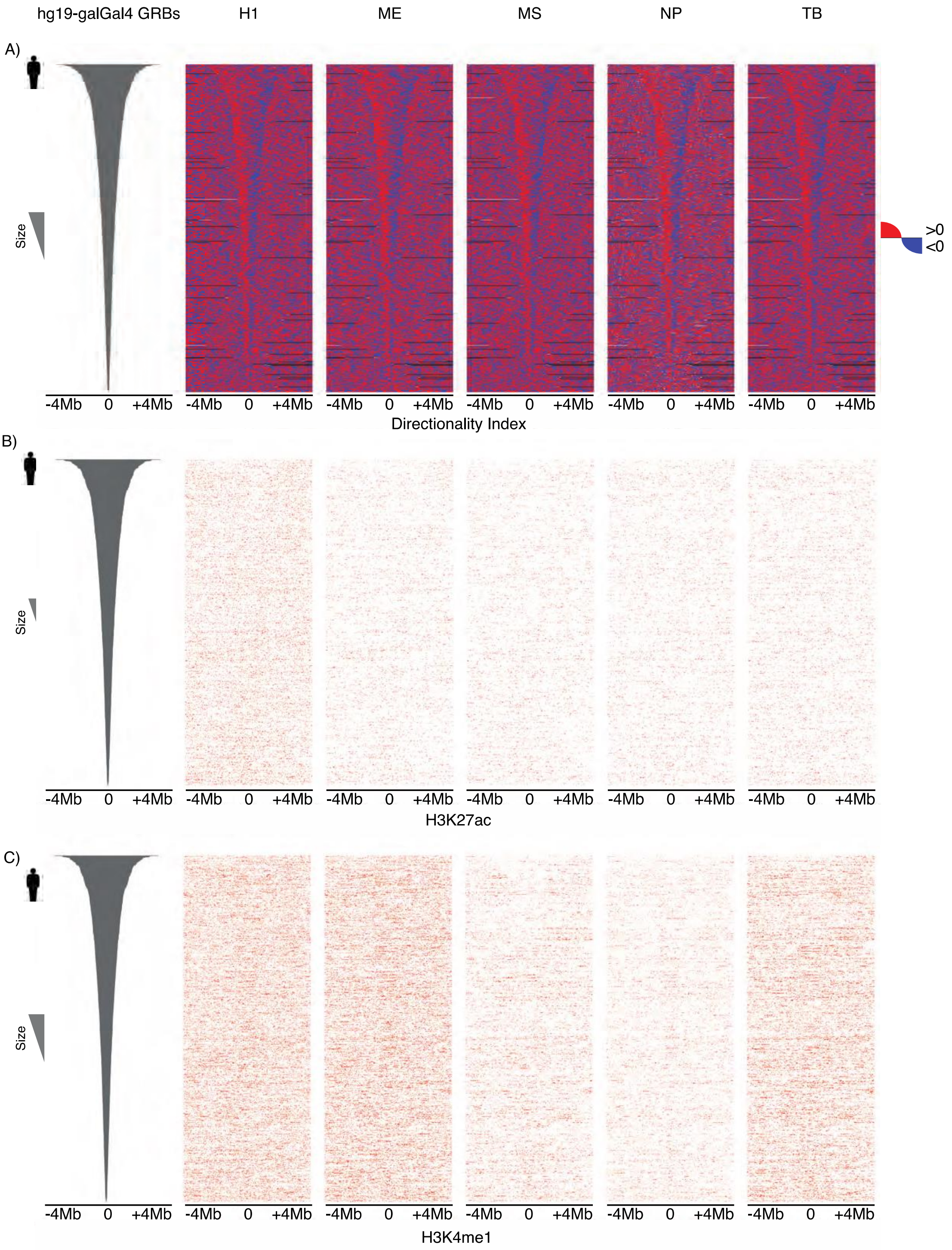

Supplementary Figure 4: Boundaries of GRBs predict topological organisation, whereas histone modification data is not predictive. A) Heatmaps representing overall direction of the Hi-C directionality index calculated in different cell lineages, spanning an 8Mb window around the centre of putative hg19-galGal4 GRBs. B) Location of H3K27ac peaks and C) H3K4me1 peaks in an 8Mb window around the centre of hg19-galGal4 GRBs reveals no obvious association between these marks and the boundaries of these regions.

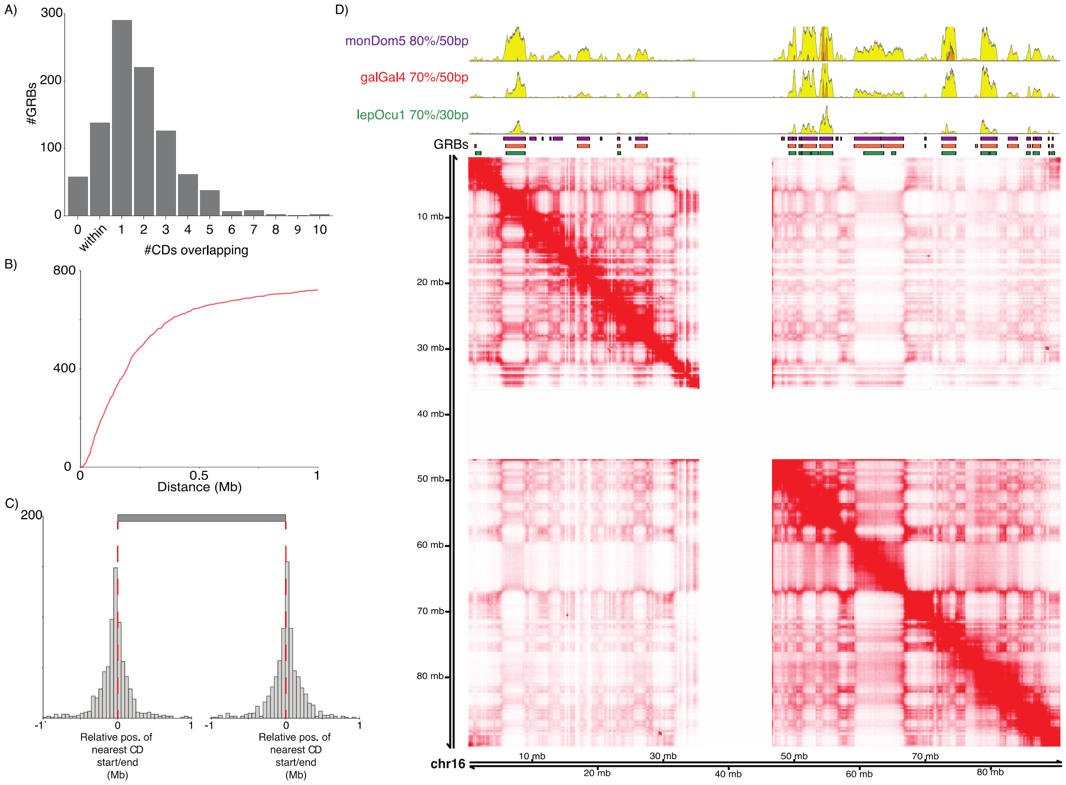

Supplementary Figure 5: The correspondence between CNE density and topological organisation is present in high-resolution Hi-C. A) Comparison of hg19-galGal4 GRBs with a set of outermost contact domains (CD) identified in Rao *et al*. identified that a large number of GRBs were located within a CD or overlapping a single CD. The large number of GRBs which are not located within a contact domain, is potentially due to the high false negative rate of the arrowhead domain finding algorithm. B) Cumulative distribution of the distance to nearest CD boundaries from GRB boundaries in GM12878 i.e. both the start and end position of a GRB lie within X kb of the nearest CD start and end. C) Relative position of the nearest CD start/end compared to the boundaries of hg19-galGal4 GRBs. D) In kilobase resolution Hi-C of GM12878 cells, the topological organisation of human chromosome 16 is highly concordant with the distribution of CNEs identified using opossum, chicken and spotted gar.

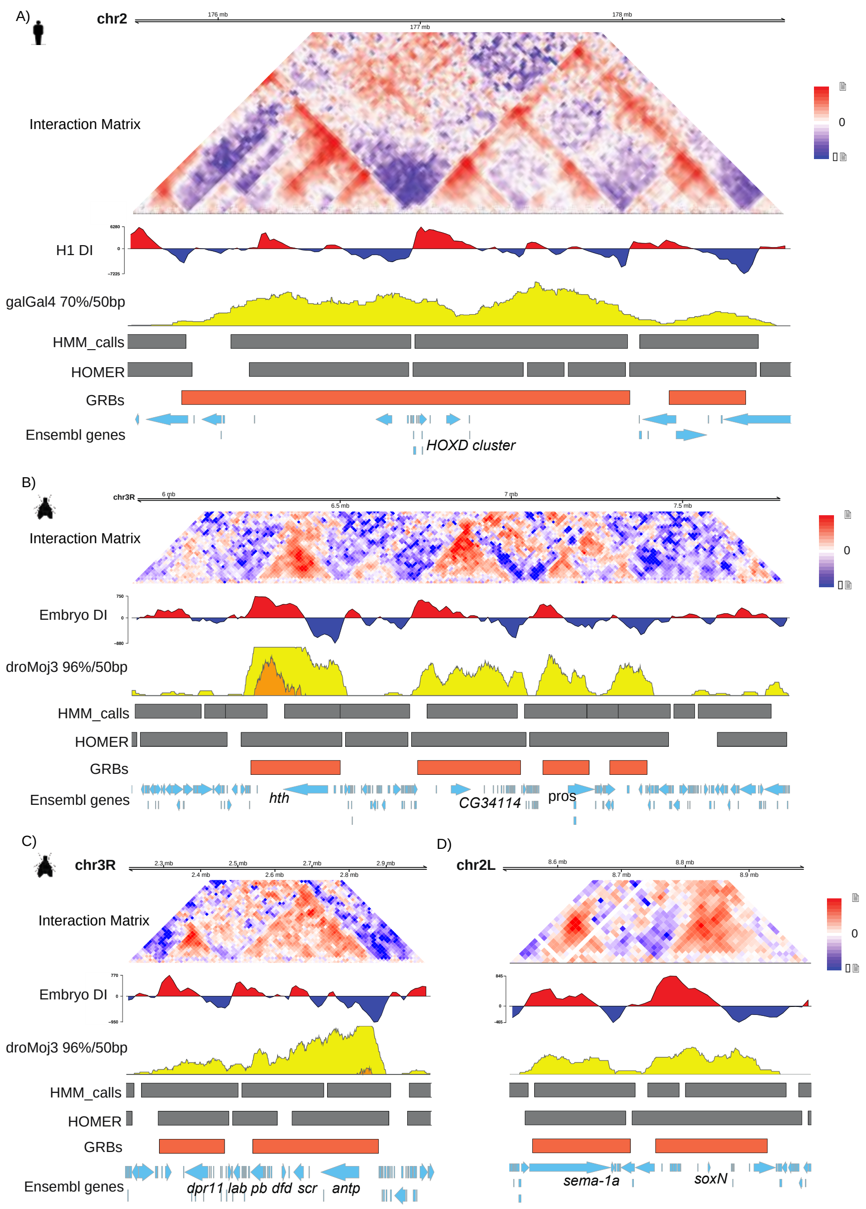

Supplementary Figure 6: Examples of genomic regulatory blocks and their associated interaction landscapes in human and *Drosophila*. GRBs at loci in both human and *Drosophila* show strong concordance with the structure of regulatory domains proposed from Hi-C. TADs, generated using both HOMER and HMM_calls, along with associated directionality index and normalised interaction matrix show a striking concordance with the boundaries of putative GRBs and with CNE density in general. A) The *HoxD* locus in human (chr2:175575640-178817020) is situated between two TADs, with its constituent genes showing interactions with regulatory elements in surrounding TADs depending on the developmental context[6-9]. The proposed *HoxD* GRB recapitulates the span of known regulatory interactions better than the regulatory domains predicted by Hi-C. B) In *Drosophila*, a region spanning (chr3R:5900280-7793200) contains *hth*, *CG34114* and *pros*, all of which have important roles in development. C) The *Drosophila* Antennapedia complex (chr3R:2198960-3016900) is one of two clusters of Hox genes in fly and contains genes necessary for the proper development of the *Drosophila* body plan. D) A region (chr2L:8510000-8990000) contains two GRBs containing the transcription factor *soxN* and the secreted transmembrane protein *sema-1a*. *soxN* is the *Drosophila* homolog of the *Sox1/2/3* transcription factors and is required for the generation of neural progenitors during development, while *sema-1a* is a neuronally expressed protein involved in regulating the localisation of axons during neurogenesis. There are a number of TADs which contain a

developmental TF but are not surrounded by a gene desert - instead, most of the GRB is spanned by target and bystander genes, with most CNEs contained in their introns. As an example, *ISL2* is a LIM homeobox located in a TAD (human chr15:76470001-77230000) which is largely covered by genes, including a long, ubiquitously expressed gene with large and numerous introns (*SCAPER*). A number of CNEs are located within these introns and microsyntenic relationship between these ISL2 and SCAPER is conserved over large evolutionary distances[10].

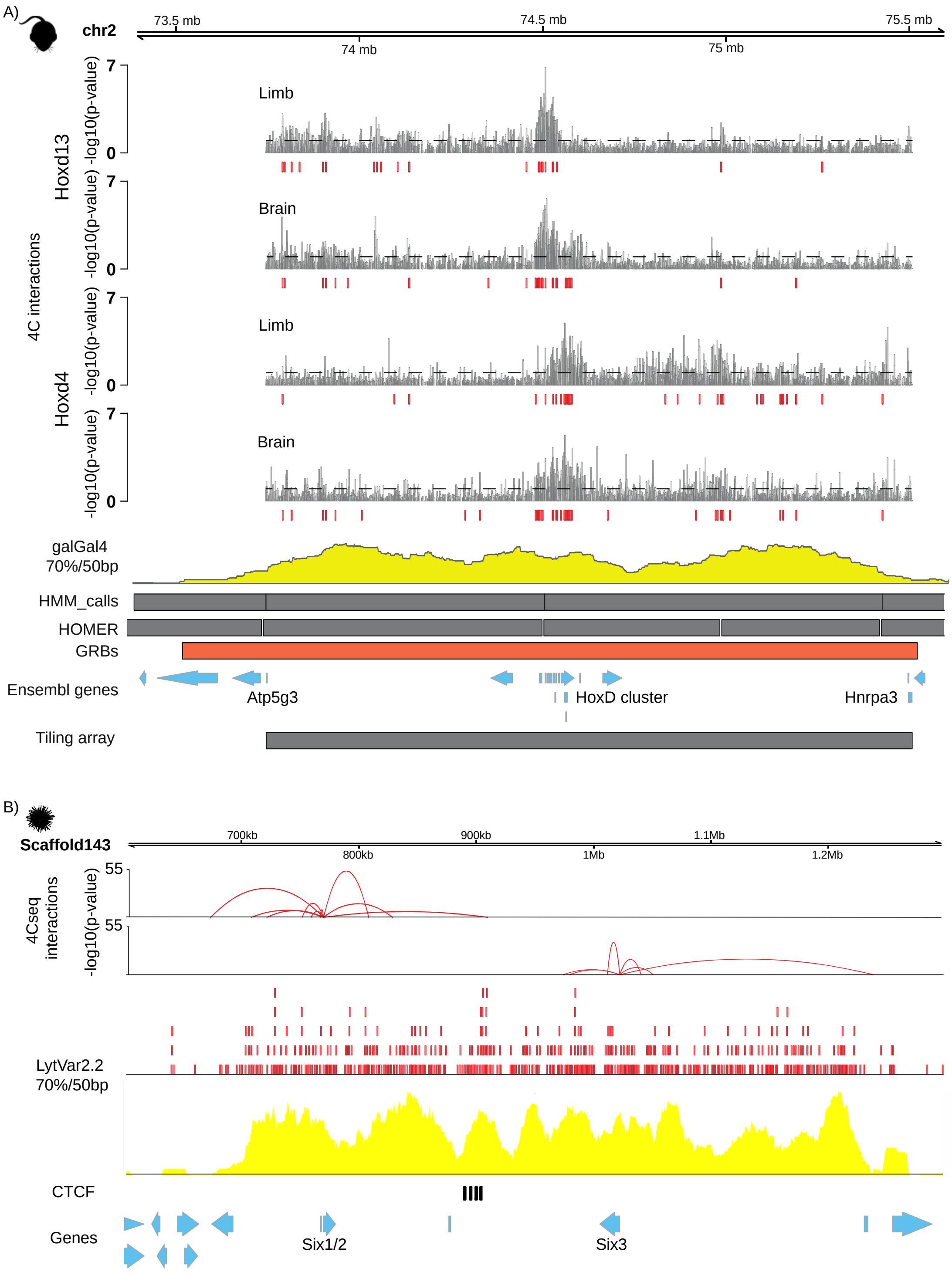

Supplementary Figure 7: The distribution of CNEs correlates with the extent of interactions at the mouse HoxD locus and sea urchin *Six* locus. A) The HoxD locus in mouse (chr2:73381800-75607400) shows the same organisation as in human, with the HoxD locus lying in the middle of two TADs, with the distribution of CNEs appearing to be highly concordant with the span of these TADs. Investigating 4C data baited at *Hoxd13* and *Hoxd4* in mouse embryonic forebrain and developing limb illustrates how *Hoxd13* and *Hoxd4* preferentially interact with either the centromeric TAD or telomeric TAD. The large syntenic

region surrounding the *HoxD* locus has previously been identified to contain large numbers of CNEs[11]. The locations of CNEs involved in significant interactions with either *Hoxd13* or *Hoxd4* are shown in red. In the developing limb *Hoxd13* interacts with 28 and 11 CNEs located with the centromeric and telomeric TADs respectively, whereas in embryonic forebrain *Hoxd13* interacts with 26 CNEs located in the centromeric TAD and 15 CNEs located within the telomeric TAD. In the developing limb *Hoxd4* interacts with 7 CNEs located within the centromeric TAD and 39 CNEs within the telomeric TAD, whereas in embryonic forebrain *Hoxd4* interacts with 23 and 35 CNEs located with the centromeric and telomeric TADs respectively. Intriguingly, CNEs are involved in interactions with the HoxD cluster in embryonic forebrain where HoxD genes are not expressed; this suggests that CNEs can be found in close proximity to their target promoter regardless of transcriptional state[12], or that CNEs are involved in their repression. This highlights the extensive roles CNEs play in the regulation of members of the HoxD cluster. B) The distribution of CNEs, identified in comparisons with *Lytechinus variegatus* (LytVar2.2), at the Six1/4/6 locus (Scaffold143:600000-1300000) in *S. purpuratus* correlates with the span of interactions identified in 4Cseq[13,14]. While it is not possible to demarcate the boundary of the regulatory domains of Six1/2 and Six3, there does appear to be local minima in CNE density close to the domain boundary defined by binding of CTCF. Data obtained from Gomez-Marin *et al.* (GSE66900). Interaction tracks visualised using GenomicInteractions[15].

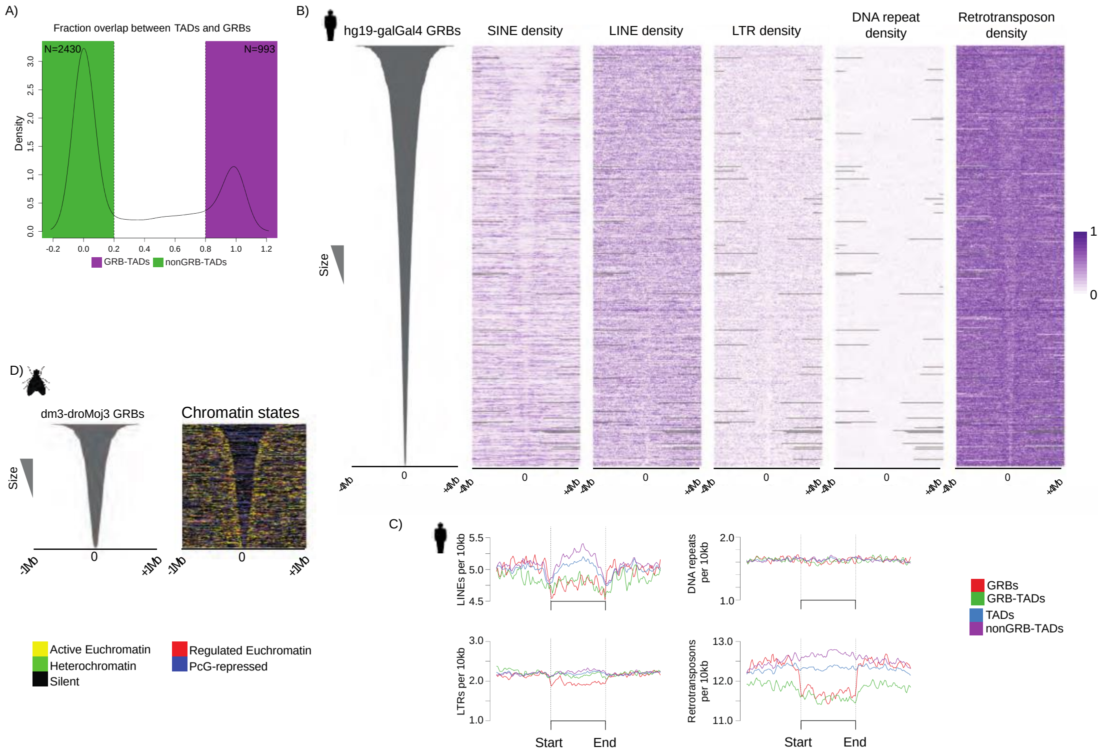

Supplementary Figure 8: Several sets of features distinguish between TADs associated with extreme non-coding conservation (GRB-TADs) from those without (nonGRB-TADs). A) Distribution of overlap of hESC HOMER TADs with putative hg19-galGal4 GRBs (70%/50bp). Thresholds for classifying TADs into GRB-TADs and nonGRB-TADs were determined based on the observed biomodality of this distribution. B) Distribution of different classes of retrotransposons in a 8Mb window centred on the midpoint of hg19-galGal4 GRBs. There is a clear depletion of SINEs within GRBs compared to surrounding regions, although there is not a depletion of other classes of retrotransposons within these regions. C) Meta-plots describing the distribution of different retrotransposon classes around the boundaries of hESC HOMER TADs and hg19-galGal4 GRBs. D) Chromatin states in a 2Mb window centred on the midpoint of dm3-droMoj3 GRBs. The vast majority of GRBs are covered primarily with *blue* and *black* chromatin, with a clear depletion in *green* and *yellow* chromatin.

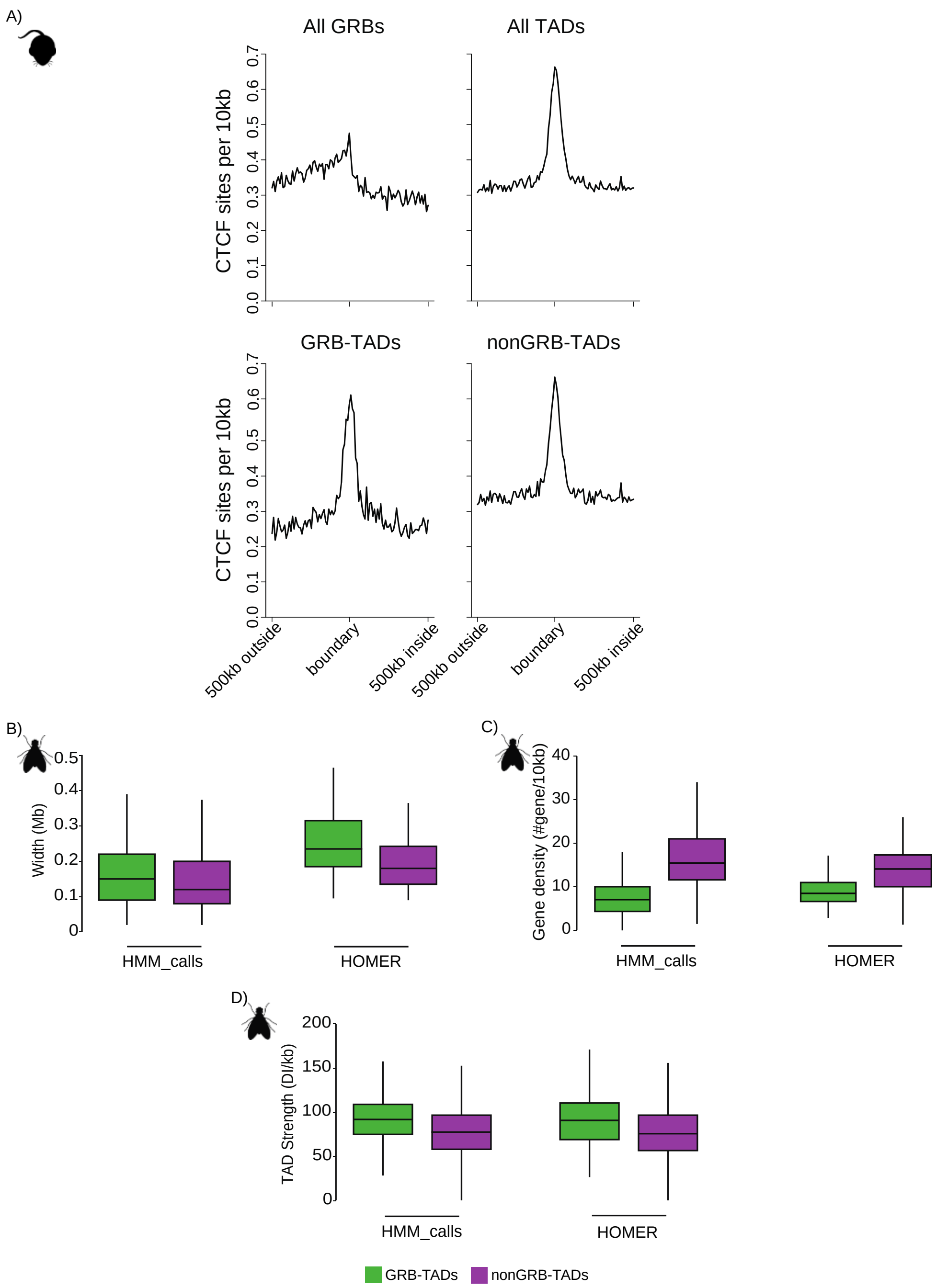

Supplementary Figure 9: Several sets of features distinguish between TADs associated with extreme non-coding conservation (GRB-TADs) from those without (nonGRB-TADs) in both mammals and flies. A) CTCF is enriched at GRB and TAD boundaries. CTCF sites per 10kb in a 1Mb window around GRB boundaries, TAD boundaries, and TADs separated into GRB-TADs and nonGRB-TADs (see Methods). B) The sizes of GRB-TADs identified in *Drosophila* whole embryos are significantly longer than nonGRB-TADs identified using either HOMER (median width 235kb vs. 185kb, $p<0.001$) or HMM_calls (median width 150kb vs. 120kb, $p=0.014$). C) *Drosophila* embryo GRB-TADs are associated with lower protein-coding gene density than nonGRB-TADs identified using either HOMER (median #genes 8.50 vs. 14.06 $p<1e-6$) or HMM_calls (median #genes 7.10 vs. 15.45 $p<1e-6$). D) Distribution of TAD strength shows that *Drosophila* GRB-TADs are significantly stronger than nonGRB-TADs identified using either HOMER (median strength 128.54 vs. 91.41, $p<1e-6$) or HMM_calls (median strength 123.08 vs. 72.95, $p<1e-6$).

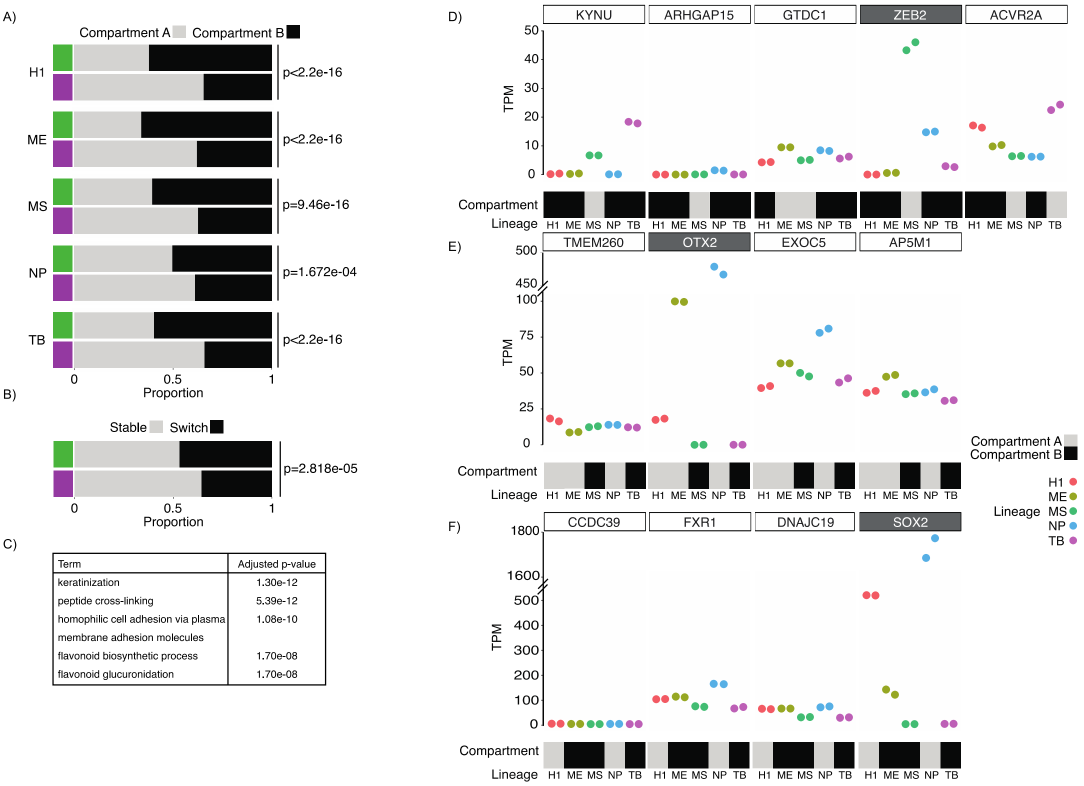

Term	Adjusted p-value
keratinization	1.30e-12
peptide cross-linking	5.39e-12
homophilic cell adhesion via plasma membrane adhesion molecules	1.08e-10
flavonoid biosynthetic process	1.70e-08
flavonoid glucuronidation	1.70e-08

Supplementary Figure 10: GRB-TADs are associated with compartment B, with changes in compartment between lineages associated with changes in expression of the GRB target gene. A) GRB-TADs (HMM_calls) are preferentially associated with Compartment B in all of the lineages investigated. B) GRB-TADs are more likely to switch compartment at least one of the five lineages investigated (i.e. A-B or B-A) than nonGRB-TADs. C) Simplified significant GO Biological process enrichment for genes located within nonGRB-TADs, but which changed compartment in at least one of the five lineages. D) A GRB (chr2:143298358-148669176) contains the developmental regulator *ZEB2* and several bystander genes. The only gene at this locus which shows dramatic upregulation in MS, when the region is now located in Compartment A is *ZEB2*. E) The GRB (chr14:56879402-57783739) containing *OTX2*, a homeobox containing TF, is located in compartment A in ME and NP concordant with an upregulation of this gene in those lineages. Nearby bystander genes do not show any large change in their expression profiles and appear to be unaffected by compartment switching. F) A GRB (chr3:180436332-182390735) contains *SOX2*, a TF important in the maintenance of pluripotency and neurogenesis. The same pattern observed at the *OTX2* and *ZEB2* loci is observed with the location of this region in Compartment A in NP and H1, largely reflecting the upregulation of *SOX2* in those lineages.

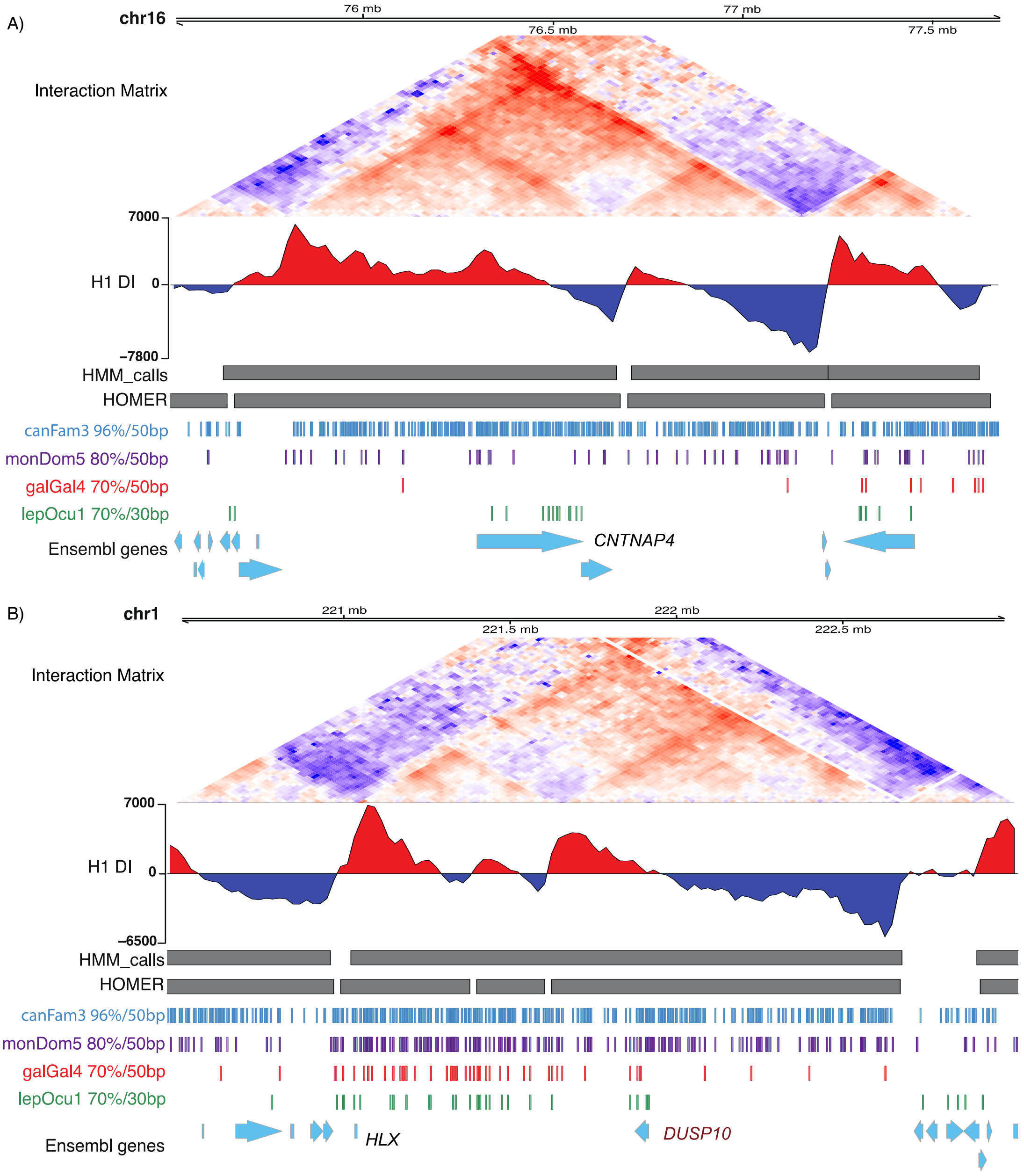

Supplementary Figure 11: Patterns of non-coding conservation provide insights into *cis*-regulatory evolution at a number of loci. A) The *CNTNAP4* locus (chr16:75500000-77690000) has a limited number of CNEs identifiable in comparisons between human and spotted gar, but lacks CNEs in comparisons between human and chicken. However, comparisons between human and opossum or dog, identifies a set of CNEs, which are predictive of the topological organisation at this locus. B) A region containing the homeobox *HLX* (chr1:220500000-223000000) shows presence of CNEs in the region from *HLX* to *DUSP10*. Although there is strong conservation of the left boundary of this region, the right boundary of this locus appears to be different dependent on the species involved. CNEs may be recruited in the region to the right of *DUSP10* suggesting potential evolutionary dynamics.

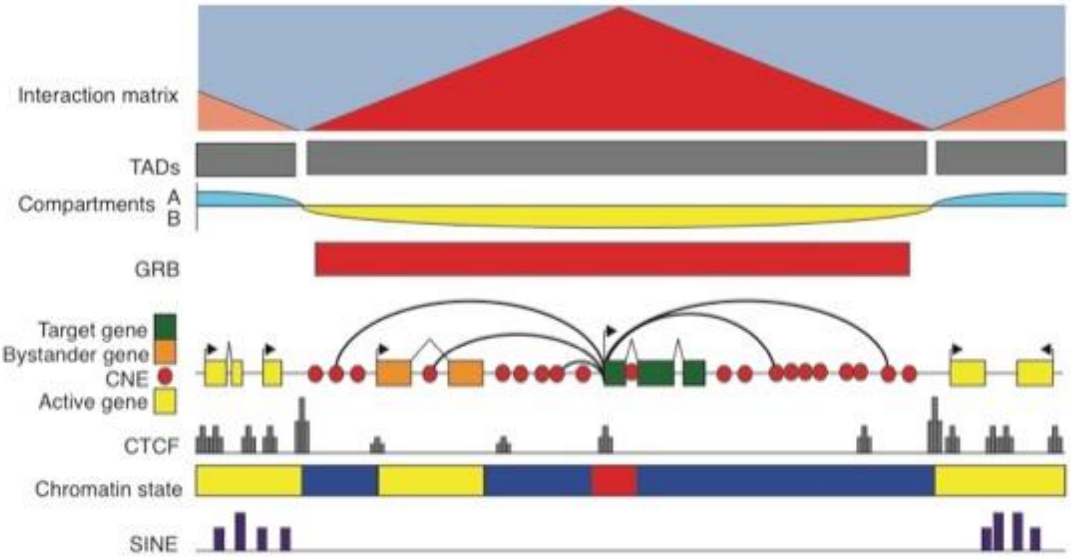

Supplementary Figure 12: Schematic for relationship of GRB-TADs, GRBs and CNEs. At loci containing important developmental regulators, the boundaries of TADs can be predicted from the distribution of CNEs. These TADs appear to be both longer and stronger than TADs lacking CNEs and are preferentially associated with compartment B. All of the regulatory elements and CNEs within this GRB-TAD are dedicated to the regulation of the GRB target gene. This regulatory domain is depleted for both CTCF and SINE elements inside it, while exhibiting enrichment for constitutive binding of CTCF at its boundaries.

Supplementary Table 1 – GRB statistics

				Summary statistics			
Genome 1	Genome 2	Percentage identity	Length	N	Minimum size	Median size	Maximum size
hg19	monDom5	80%	50bp	1159	17612	676245	9206749
hg19	galGal4	90%	50bp	4965	97593	1388520	8678549
hg19	galGal4	80%	50bp	667	10049	1047838	7907716
hg19	galGal4	70%	50bp	816	10118	881448	7222385
hg19	lepOcu1	70%	30bp	715	1606	562873	3956612
mm9	galGal4	70%	50bp	774	10380	690086.5	5697791
dm3	droMoj3	96%	30bp	317	7878	127687	714028

Supplementary Table 2 – Correspondence between GRB and TAD boundaries

GRB set	Cell line	Method	Median relative distance from GRB start to nearest TAD boundary (bp)	Median relative distance from GRB end to nearest TAD boundary (bp)	Median absolute distance from GRB start to nearest TAD boundary (bp)	Median absolute distance from GRB end to nearest TAD boundary (bp)	# GRBs with both edges < 120kb	p-value
hg19/galGal4 (70%/50bp)	H1	HOMER	-54214	62076	112002	112895	235	p<1e-5
	H1	DIXON	-109246	98235	183483.5	174008	129	p<1e-5
	Mesenchymal	HOMER	-56984	57845.5	112716.5	106477	233	p<1e-5
	Mesenchymal	DIXON	-104081	111511.5	168057.5	185671.5	126	p<1e-5
	Mesendoderm	HOMER	-70351	70523	124723	125357	196	p<1e-5
	Mesendoderm	DIXON	-154119	106745	222259	184264	110	p<1e-5
	NP	HOMER	-66051.5	66415.5	117730.5	118858	209	p<1e-5
	NP	DIXON	-167250	135056	236494	228637	103	p<1e-5
	TB	HOMER	-63603	65680	122858.5	115187.5	198	p<1e-5
	TB	DIXON	-109598	122154	183730	187024	128	p<1e-5
hg19/monDom5 (80%/50	H1	HOMER	-54844.5	57989.5	112511	114573.5	314	p<1e-5
	H1	DIXON	-112127	120208	181416	185362	190	p<1e-5
	Mesenchymal	HOMER	-60102	53881	109767	106257	329	p<1e-5
	Mesenchymal	DIXON	-104835	120208	169820	195725	183	p<1e-5
	Mesendoderm	HOMER	-54658	74262.5	120744.5	120119.5	279	p<1e-5
	Mesendoderm	DIXON	-143499	131804	206497	199548	168	p<1e-5
	NP	HOMER	-66009	54426	112018	112663	306	p<1e-5
	NP	DIXON	-184474.5	168087	240960	233378	148	p<1e-5
	TB	HOMER	-61200	60638	118896	118454	305	p<1e-5
	TB	DIXON	-103591	134548	161497	201526	189	p<1e-5
hg19/lepOcu1 (70%/30bp	H1	HOMER	-68548	56677	145198	136693	141	p<1e-5
	H1	DIXON	-185361	163029	246625	223948	65	p<1e-5
	Mesenchymal	HOMER	-59418	52081	138839	127798	150	p<1e-5
	Mesenchymal	DIXON	-166586	149047	237379	219458	69	p<1e-5
	Mesendoderm	HOMER	-87691	80400.5	165592.5	162916.5	113	p<1e-5
	Mesendoderm	DIXON	-232507	179720.5	288766	242825.5	62	p<1e-5
	NP	HOMER	-59530	70135	145972	146757	123	p<1e-5
	NP	DIXON	-275540	258653	327739.5	306696	48	p<1e-5
	TB	HOMER	-64126.5	63414	150838.5	141514.5	126	p<1e-5
	TB	DIXON	-180310	162106	243246	233226	69	p<1e-5

	Cell line	Method	Median relative distance from GRB start to nearest TAD boundary (bp)	Median relative distance from GRB end to nearest TAD boundary (bp)	Median absolute distance from GRB start to nearest TAD boundary (bp)	Median absolute distance from GRB end to nearest TAD boundary (bp)	# GRBs with both edges < 40kb	p-value
dm3/droMoj3 96%/50bp	dEmbryo	HOMER	-20095	27960.5	26668.5	33294.5	102	p<1e-5
	dEmbryo	DIXON	-20887	18617	29260	29116	106	p<1e-5

Supplementary Table 3 – TAD statistics

			hg19-monDom5 (90%/50bp)		hg19-galGal4 (90%/50bp)		hg19-galGal4 (80%/50bp)		hg19-galGal4 (70%/50bp)		hg19-lepOcu1 (70%/30bp)	
Cell line	Method	N	#GRB-TADS	#NONGRB-TADS	#GRB-TADS	#NONGRB-TADS	#GRB-TADS	#NONGRB-TADS	#GRB-TADS	#NONGRB-TADS	#GRB-TADS	#NONGRB-TADS
H1	HOMER	4034	1144	2025	903	2779	1015	2521	993	2430	467	3031
H1	DIXON	2698	582	1316	482	1847	526	1643	511	1576	207	1969
Mesenchymal	HOMER	3463	887	1758	707	2423	796	2185	777	2106	362	2588
Mesenchymal	DIXON	2345	455	1132	390	1619	424	1428	407	1366	171	1702
Mesendoderm	HOMER	4011	1125	2012	882	2776	983	2522	966	2421	451	3021
Mesendoderm	DIXON	2744	589	1343	492	1886	528	1685	504	1618	212	2013
NP	HOMER	3558	1020	1719	803	2402	889	2152	873	2062	407	2630
NP	DIXON	1923	393	811	352	1221	371	1051	352	1004	137	1325
TB	HOMER	3689	994	1861	790	2570	879	2326	858	2234	403	2759
TB	DIXON	2649	577	1282	491	1813	527	1609	502	1541	201	1928

			mm9-galGal4 (70%/50bp)	
Cell line	Method	N	#GRB-TADS	#NONGRB-TADS
mESC	HOMER	3747	694	2478
mESC	DIXON	2200	311	1352
mCortex	HOMER	3145	608	2032
mCortex	DIXON	1519	181	849

			dm3-droMoj3 (96%/50bp)	
Cell line	Method	N	#GRB-TADS	#NONGRB-TADS
dEmbryo	HOMER	357	136	106
dEmbryo	DIXON	489	122	211

Supplementary Methods

Generating the null distribution of GRB-TAD edge distances

The significance of the number of GRBs having both edges within a specified distance of a TAD boundary was calculated by comparing the observed number of GRBs with that observed by randomly shuffling GRBs. A null distribution was created by generating 100000 shuffled regions using BEDtools[16], excluding centromeric regions, and calculating the number of random regions whose boundaries were within Xkb bins of the nearest TAD boundary. A distance of 120kb was used for all comparisons involving human and 40kb for comparisons involving *Drosophila*.

Histone modification data

H3K4me1 and H3K27ac ChIP-seq data for human H1, ME, MS, NP and TB cells was obtained from GSE16256[17], and aligned against hg19 using bowtie[18]. Enriched regions were identified using MACS2[19], using default parameters. ChIP dataset quality was assessed using ChIPQC[20], and the dataset with the highest ChIP enrichment was used (identified as the replicate having the maximum RiP (reads in peaks)). In order to prevent promoters marked by H3K27ac from affecting our analyses, H3K27ac signal present within 2.5kb of Ensembl annotated TSSs was removed. The location of significantly enriched regions within 5kb bins was then calculated in an 8Mb window around the midpoint of putative GRBs and visualised.

Analysis of 4C data

4C data was obtained from GSE31659[7]. Annotation for the tiling array was lifted over from mm8 to mm9. Datasets were normalised and processed using the MRA.TA package at the level of restriction enzyme fragments[21] and p-values combined across replicates using Fisher's method. Those interactions with a p-value < 0.1 were classified as significant interactions. A CNE was classified as interacting with *Hoxd13* or *Hoxd4* if it overlapping a restriction fragment that was involved in an interaction (p-value < 0.1) with either of these two genes.

RNA-seq analysis

RNA-seq data was obtained from GSE16256[22], and aligned against Ensembl genes release 75 using Tophat2[23]. Aligned reads were counted using htseq[24]. Differential expression analysis was performed using DESeq2[25].

Supplementary References

1. Kikuta, H. *et al.* Genomic regulatory blocks encompass multiple neighboring genes and maintain conserved synteny in vertebrates. *Genome Res.* **17,** 545–555 (2007).
2. Engström, P. G., Ho Sui, S. J., Drivenes, O., Becker, T. S. & Lenhard, B. Genomic regulatory blocks underlie extensive microsynteny conservation in insects. *Genome Res.* **17,** 1898–1908 (2007).
3. Pennacchio, L. A. *et al.* In vivo enhancer analysis of human conserved non-coding sequences. *Nature* **444,** 499–502 (2006).
4. Royo, J. L. *et al.* Dissecting the Transcriptional Regulatory Properties of Human Chromosome 16 Highly Conserved Non-Coding Regions. *PLoS ONE* **6,** e24824 (2011).
5. Navratilova, P. *et al.* Systematic human/zebrafish comparative identification of cis-regulatory activity around vertebrate developmental transcription factor genes. *Dev. Biol.* **327,** 526–540 (2009).
6. Morey, C., Da Silva, N. R., Perry, P. & Bickmore, W. A. Nuclear reorganisation and chromatin decondensation are conserved, but distinct, mechanisms linked to Hox gene activation. *Development* **134,** 909–919 (2007).
7. Montavon, T. *et al.* A regulatory archipelago controls Hox genes transcription in digits. *Cell* **147,** 1132–1145 (2011).
8. Noordermeer, D. *et al.* The dynamic architecture of Hox gene clusters. *Science* **334,** 222–225 (2011).
9. Williamson, I. *et al.* Anterior-posterior differences in HoxD chromatin topology in limb development. *Development* **139,** 3157–3167 (2012).
10. Irimia, M. *et al.* Extensive conservation of ancient microsynteny across metazoans due to cis-regulatory constraints. *Genome Res.* **22,** 2356–2367 (2012).
11. Lee, A. P., Koh, E. G. L., Tay, A., Brenner, S. & Venkatesh, B. Highly conserved syntenic blocks at the vertebrate Hox loci and conserved regulatory elements within and outside Hox gene clusters. **103,** 6994–6999 (2006).
12. Ghavi-Helm, Y. *et al.* Enhancer loops appear stable during development and are associated with paused polymerase. *Nature* **512,** 96–100 (2014).
13. Cameron, R. A., Samanta, M., Yuan, A., He, D. & Davidson, E. SpBase: the sea urchin genome database and web site. *Nucleic Acids Res* **37,** D750–4 (2009).
14. Gómez-Marín, C. *et al.* Evolutionary comparison reveals that diverging CTCF sites are signatures of ancestral topological associating domains borders. **112,** 7542–7547 (2015).
15. Harmston, N., Ing-Simmons, E., Perry, M., Baresic, A. & Lenhard, B. GenomicInteractions: An R/Bioconductor package for manipulating and investigating chromatin interaction data. *BMC Genomics* **16,** 963 (2015).
16. Quinlan, A. R. & Hall, I. M. BEDTools: a flexible suite of utilities for comparing genomic features. *Bioinformatics* **26,** 841–842 (2010).
17. Roadmap Epigenomics Consortium *et al.* Integrative analysis of 111 reference human epigenomes. *Nature* **518,** 317–330 (2015).
18. Langmead, B., Trapnell, C., Pop, M. & Salzberg, S. L. Ultrafast and memory-efficient alignment of short DNA sequences to the human genome. *Genome Biol.* **10,** R25 (2009).

19. Zhang, Y. *et al.* Model-based analysis of ChIP-Seq (MACS). *Genome Biol.* **9,** R137 (2008).
20. Carroll, T. S., Liang, Z., Salama, R., Stark, R. & de Santiago, I. Impact of artifact removal on ChIP quality metrics in ChIP-seq and ChIP-exo data. *Front Genet* **5,** 75 (2014).
21. Leblanc, B., Comet, I., Bantignies, F. & Cavalli, G. Chromosome Conformation Capture on Chip (4C): Data Processing. *Methods Mol. Biol.* **1480,** 243–261 (2016).
22. Xie, W. *et al.* Epigenomic analysis of multilineage differentiation of human embryonic stem cells. *Cell* **153,** 1134–1148 (2013).
23. Kim, D. *et al.* TopHat2: accurate alignment of transcriptomes in the presence of insertions, deletions and gene fusions. *Genome Biol.* **14,** (2013).
24. Anders, S., Pyl, P. T. & Huber, W. HTSeq--a Python framework to work with high-throughput sequencing data. *Bioinformatics* **31,** 166–169 (2015).
25. Love, M. I., Huber, W. & Anders, S. Moderated estimation of fold change and dispersion for RNA-seq data with DESeq2. *Genome Biol.* **15,** 550 (2014).
